# P-1929. Impact of the COVID-19 Pandemic on the Risk Factors and Outcomes Associated with Candidemia

**DOI:** 10.1093/ofid/ofae631.2089

**Published:** 2025-01-29

**Authors:** Melat Endashaw, Austin Dykes, Rachael Clark, Laila Warrayat, Shiyuan Deng, Xianyan Chen, Andrés F Henao Martínez, Daniel B Chastain

**Affiliations:** Emory Healthcare / University of Georgia College of Pharmacy, Tucker, Georgia; Baptist Medical Center, Jacksonville, Florida; University of Georgia College of Pharmacy, Cumming, Georgia; University of Georgia College of Pharmacy, Cumming, Georgia; University of Georgia, Athens, Georgia; UGA Franklin College of Arts and Sciences, Athens, Georgia; University of Colorado Anschutz Medical Campus, Aurora, Colorado; University of Georgia College of Pharmacy, Cumming, Georgia

## Abstract

**Background:**

Cases of candidemia surged during the COVID-19 pandemic, possibly due to the intensive treatments used in critically ill patients. This study aims to explore the impact of the pandemic on risk factors and clinical outcomes associated with candidemia.

**Figure:**
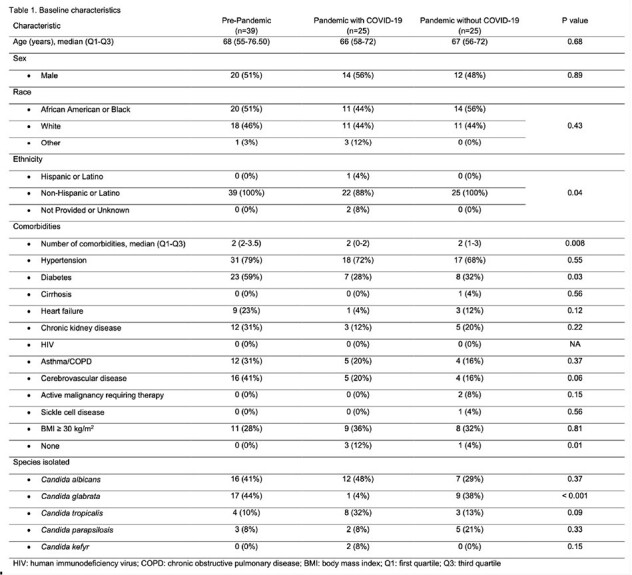

**Methods:**

We retrospectively analyzed patients ≥ 18 years diagnosed with candidemia at a single facility in Albany, Georgia, between January 2017 and May 2023. Patients were categorized into three groups based on their COVID-19 status and admission date (January 21, 2020, marking the first US case): 1) pre-pandemic (before January 21, 2020), 2) pandemic with COVID-19 (after January 21, 2020), and 3) pandemic without COVID-19 (after January 21, 2020). We compared risk factors for candidemia and in-hospital mortality across these groups.

**Figure:**
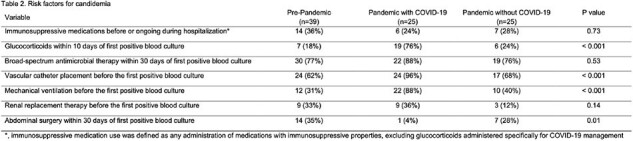

**Results:**

A total of 89 patients were included, with 44% pre-pandemic and 56% during the pandemic (half with COVID-19) (Table 1). Baseline characteristics were similar, except for a higher median number of comorbidities in the pre-pandemic group (p=0.008). Notably, the COVID-19 group had a significantly higher proportion of patients with no comorbidities (12%) compared to the pre-pandemic (0%) and pandemic without COVID-19 (4%) groups (p=0.01). Patients with COVID-19 were predominantly diagnosed with candidemia in the ICU (92%) compared to pre-pandemic (49%) and pandemic without COVID-19 (44%) groups (p< 0.001). Additionally, mechanical ventilation and vascular catheters were more frequent in the COVID-19 group (88% and 96%, respectively) compared to the pre-pandemic (31% and 62%) and pandemic without COVID-19 (40% and 68%) groups (p< 0.001 for both) (Table 2). While immunosuppressive medications were uncommon, glucocorticoid use was significantly higher in the COVID-19 group (76%) compared to the pre-pandemic (18%) and pandemic without COVID-19 (24%) groups (p=0.008). In-hospital mortality was highest among patients with COVID-19 (76%) compared to patients before the pandemic (44%) and those without COVID-19 during the pandemic (32%) (p< 0.005).

**Conclusion:**

Patients with COVID-19 displayed a higher prevalence of candidemia risk factors and significantly worse clinical outcomes, including higher in-hospital mortality, compared to the other groups.

**Disclosures:**

All Authors: No reported disclosures

